# *In vitro *evaluation of HMGB1 removal with various membranes for continuous hemofiltration

**DOI:** 10.1186/cc9534

**Published:** 2011-03-11

**Authors:** M Yumoto, O Nishida, K Moriyama, Y Shimomura, T Nakamura, N Kuriyama, Y Hara, S Yamada, T Miyasho

**Affiliations:** 1 Fujita Health University School of Medicine, Toyoake, Japan; 2 Shino-test Corporation, Sagamihara, Japan; 3 Rakuno Gakuen University, Ebetsu, Japan

## Introduction

The high mobility group box 1 protein (HMGB1) is an alarmin that plays an important role in sepsis. HMGB1 is hardly removable by normal hemofiltration because of its large molecular weight of 30 kDa. Here we show the possibility of removing HMGB1 from the blood.

## Methods

The test solution contained 100 μg HMGB1 and 35 g albumin in 1,000 ml of a substitution fluid. Experimental hemofiltration (solution flow of 100 ml/minute and ultrafiltrate flow of 1,000 ml/hour) was conducted for 360 minutes in a closed loop circulation system, and the sieving coefficient (SC) and ultrafiltrate and blood clearance rates of HMGB1 were calculated. High cut-off (HCO), AN69ST, polysulfone (PS), and polymethylmethacrylate (PMMA) membranes were tested (*n *= 4).

## Results

**Figure 1:**
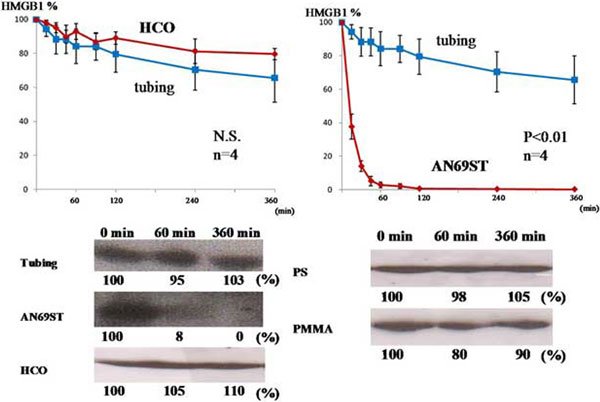
**Percentage of HMGB1 remaining in the test solutions with HCO and AN69ST membrane**.

The concentrations (means ± SD) of HMGB1 at 0, 60 and 360 minutes of hemofiltration for AN69ST (74.0 ± 11.8, 2.1 ± 1.2, and 0.5 ± 0.6 ng/ml) decreased significantly by adsorption. Relative concentrations of HMGB1 as determined by western blot analysis and the calculated clearance rates were obtained. Among the four membranes, AN69ST showed the highest capacity to adsorb HMGB1; it adsorbed 100 μg of HMGB1 in the initial 60 minutes and showed a markedly high clearance (60.8 ± 5.0 ml/minute) at 15 minutes. Although the highest SC for HMGB1 was 0.7 with the HCO membrane, which correlated with a constant filtrate clearance rate, albumin loss was observed. No such removal of both HMGB1 and albumin was observed with the PS membrane and tubing. See Figure 1.

## Conclusions

Continuous hemofiltration using HCO or AN69ST membrane will be a promising approach for HMGB1-related sepsis.
